# Health Tract, No. 115

**Published:** 1862-11

**Authors:** 


### KEEPING APPLES.

The apple is the most valuable of all the fruits of the earth, in consequence of its lusciousness, its preservability, the variety of uses to which it can be applied, and its productiveness. Mr. E. Lake, of Topsfield, Massachusetts, in 1861 obtained, from one acre of ground, two hundred barrels of Baldwin Russet apples, besides a ton and a half of squashes and a hundred cabbages; one weighing twenty-seven pounds. It has been stated that a single tree has yielded in one season forty bushels of apples. A hundred and fifty good-sized apples make a bushel. Two baked apples are an abundant desert for dinner; two each for breakfast and supper, with a single cup of tea or coffee, and as much bread and butter as is wanted, is as much as children and sedentary persons ought to have. Apples come in August and keep good until May, nine months, two hundred and seventy days, fifteen hundred apples, or ten bushels, or four barrels; easily had in the country for one dollar each. Thus four dollars' worth of apples will furnish one person, three parts of the year, with a "relish" for breakfast and supper, and "dessert" for dinner, of which he will no more "get tired" than of bread, and it will be cheaper and incomparably more healthful than pies, preserves, sweetmeats, doughnuts, cookies, dumplings, puddings, and the other long list of stomach-destroying and dyspepsia-engendering articles.

Preserving Apples.—Pick out the perfect ones, pack them away, surrounding each apple with dry, ground plaster of Paris. Thus: begin with an inch of plaster, then a layer of apples an inch from the side, and half an inch apart; sift in the plaster until covered nearly an inch, and so on until the receptacle is full. This fertilizing plaster costs from three to ten dollars a ton, and is as good in the spring for such purpose as if it had not been used. Pippins will keep until June in any cool, dry room in the house.

Apples, spread on a board-floor, and covered with five or six layers of newspapers, or a sheet, or clean straw, will keep until spring, and even on a common dry cellar-floor. Apples will keep many months if, free from any bruise or speck, they are wrapped each in soft paper and laid on a shelf cool and dry. In cities and towns, apples, as commonly bought in barrels, will keep pretty well until spring in a dry cellar; but they should be carefully picked over, and the unspecked ones laid down softly every two weeks. Even laid on shelves, two layers deep, and covered with newspapers or straw, picking out the specked ones for use, every few days, very few will be lost. Very good apples can be bought in New-York, during the latter part of October, for one dollar and a half a barrel; and if cared for and used as above, and in addition given out to school-children for luncheon, instead of nuts, sweet-cakes, candy, cross-buns, doughnuts, and the like, sickness would be prevented, and money saved to an amount which would surprise any one who never "tried it." Those who live in the country will save themselves a great deal of trouble, and admirably succeed in keeping apples in perfect order until June, if they would take the precaution to pick each apple from the tree and lay it in a basket; then lay them on the floor of a cool, dry room for a few days to dry, and then pack them away in some one of the plans above suggested. It ought to be known that a baked sweet apple is the most digestible food that can be swallowed; they are digested in about an hour and a half, bread requiring two hours longer. Let invalids remember this.

